# District and sub-district analysis of cryptococcal antigenaemia prevalence and specimen positivity in KwaZulu-Natal, South Africa

**DOI:** 10.4102/ajlm.v7i1.757

**Published:** 2018-10-11

**Authors:** Naseem Cassim, Lindi M. Coetzee, Nelesh P. Govender, Deborah K. Glencross

**Affiliations:** 1 Department of Haematology and Molecular Medicine, University of the Witwatersrand, Johannesburg, South Africa; 2 National Priority Programme, National Health Laboratory Service, Johannesburg, South Africa; 3 National Institute for Communicable Diseases, Division of the National Health Laboratory Service (NHLS), Johannesburg, South Africa; 4 Faculty of Health Sciences, University of the Witwatersrand, Johannesburg, South Africa

## Abstract

**Background:**

Cryptococcal meningitis (CM) is a leading cause of mortality among HIV-positive South Africans. Reflex cryptococcal antigen (CrAg) testing of remnant plasma was offered as a pilot prior to implementation in October 2016 in KwaZulu-Natal province. The national reflex CrAg positivity was 5.4% compared to 7.3% for KwaZulu-Natal.

**Objectives:**

The aim of this study was to interrogate CrAg positivity by health levels to identify hotspots.

**Method:**

Data for the period October 2016 to June 2017 were analysed. Health district CrAg positivity and prevalence were calculated, with the latter using de-duplicated patient data. The district CrAg positivity and the number of CrAg-positive specimens per health facility were mapped using ArcGIS. For districts with the highest CrAg positivity, a sub-district CrAg positivity analysis was conducted.

**Results:**

The provincial CrAg positivity was 7.6%. District CrAg positivity ranged from 5.7% (Ugu) to 9.6% (Umkhanyakude) with prevalence ranging from 5.5% (Ugu) to 9.7% (Umkhanyakude). The highest CrAg positivity was reported for the Umkhanyakude (9.6%) and King Cetswayo (9.5%) districts. In these two districts, CrAg positivity of 10% was noted in the Umhlabuyalingana (10.0%), Jozini (10.2%), uMhlathuze (10.5%) and Nkandla (10.8%) subdistricts. In these subdistricts, 135 CrAg-positive samples were reported for the Ngwelezane hospital followed by 41 and 43 at the Hlabisa and Manguzi hospitals respectively.

**Conclusion:**

Cryptococcal antigen positivity was not uniformly distributed at either the district or sub-district levels, with identified facility hotspots in the Umkhanyakude and King Cetswayo districts. This study demonstrates the value of laboratory data to identify hotspots for planning programmatic interventions.

## Introduction

Cryptococcal meningitis (CM) is a leading cause of mortality among HIV-positive persons in South Africa.^1^ Cryptococcal antigenaemia screening can identify persons at risk of developing CM (even among asymptomatic patients) to timeously initiate antifungal and antiretroviral treatment (ART) for naive persons. Cryptococcal antigen (CrAg) reflex testing of specimens with a CD4 count of ≤ 100 cells/µl was implemented across a network of CD4 testing laboratories in South Africa.^2^ This followed the inclusion of CrAg screening in South Africa’s national HIV guidelines in 2015, following recommendations by the World Health Organization (WHO).^3,4^

Since the national implementation of reflex CrAg screening in South Africa in October 2016, data collected by testing laboratories confirmed that nationally, about 10% of all CD4 samples tested had a count ≤ 100 cells/µl. These specimens were tested for CrAg using a lateral flow assay (LFA) (Immuno Mycologics, Norman, Oklahoma, United States). Both the percentage of samples eligible for CrAg testing and the percentage of CrAg-positive results vary considerably across the nine provinces in South Africa. Data from a previously reported study showed a CrAg positivity of 2.4% in the Northern Cape versus 7.3% in KwaZulu-Natal.^5^ Within KwaZulu-Natal, district CrAg positivity ranged from 6.2% in Amajuba to 9.2% in King Cetshwayo district, with the Umkhanyakude district reporting the second highest CrAg positivity at 8.9%.^5^

KwaZulu-Natal is the second most populous province in South Africa and has the highest HIV prevalence.^6^ There are 11 079 700 people living in the province representing 19.8% of the national population.^7^ The provincial HIV prevalence is 16.9% in 2016 compared to the national prevalence of 12.2%. The 2016 HIV infection rate in KwaZulu-Natal is 2.3% (compared to the national rate of 1.8%^6^) among 1 622 870 million HIV-seropositive individuals (15–49 years of age) living in the province. An estimated 1 129 314 HIV-positive individuals are on ART with 69.5% coverage.^6^ Reflex CrAg screening was offered for the first time in KwaZulu-Natal at the Prince Mshyeni Memorial laboratory in July 2015 as part of a CrAg reflex testing pilot.^8,9^ By October 2016, all CD4 laboratories in KwaZulu-Natal offered reflex CrAg screening.

There are limited published CM data available for KwaZulu-Natal as most reported studies investigated selected health facilities with small case numbers. A study conducted at an urban district hospital between 2011 and 2012 reported that of the 127 patients with confirmed CM, 65 were men (51.2%).^10^ In that study, CM affected predominantly the economically active population (mean age, 36 [±9.8] years).^10^ While 76% (*n* = 97) of patients knew their HIV status, but only 45% (*n* = 43) were on ART.^10^ Acute mortality was 55.9% (71/127) within 14 days of CM diagnosis highlighting the need for screening and preemptive treatment of subclinical cryptococcal disease before ART initiation.^10^ A 2007 study conducted at the Ngewlezane hospital reported that even in a setting where amphotericin B is available, the burden of CM deaths is particularly high in the immediate period after diagnosis (2.13 deaths per 100 person days).^11^ Of the 186 patients enrolled in this cohort, 52 (28%) died within 14 days of diagnosis.^11^ In 2015 the National Institute for Communicable Diseases reported 1745 laboratory-confirmed cases of CM from KwaZulu-Natal through its national network of clinical microbiology laboratories which participate in an active surveillance programme for pathogens of public health importance (GERMS-SA).^12^

Given the higher-than-national average CrAg positivity in KwaZulu-Natal, the study reported here aimed to investigate CrAg positivity at the health district and sub-district levels with facility level investigation in the subdistricts with the highest reported positivity rates.

## Methods

### Ethical considerations

Ethics clearance for this work was obtained from the University of the Witwatersrand (study approval number M1706108). This study was conducted in accordance with relevant national and international guidelines. This study involved the secondary analysis of laboratory test volumes data that do not contain any patient identifiers. No patient recruitment was necessary as routine laboratory data was used for the study.

### Data

Data reported here include reflex CrAg testing subsequent to a confirmed CD4 count ≤ 100 cells/µl using automated laboratory information system rules that identify eligible samples to be tested, that is, these data exclude provider-initiated CrAg screening. Owing to CD4 testing challenges at the Church of Scotland laboratory, all CrAg data from this laboratory were excluded for the purpose of this study. This laboratory serves the Umzinyathi health district that contributes only 22% of CrAg samples tested in this district.

The data analysis includes both CrAg positivity using specimen-level data as well as CrAg prevalence after the data set was de-duplicated. The de-duplication process involved using the Corporate Data Warehouse (CDW)-assigned unique patient identifier that is determined through a probabilistic matching algorithm to identify if a patient had more than one CrAg test.^13^ The de-duplicated data were used to identify both the number of patients receiving a CrAg test as well as the number with one or more positive CrAg tests to determine prevalence.^13^ The de-duplicated data provide a more accurate analysis of CrAg positivity for a given population.

### Data extraction

CD4 and CrAg laboratory data were extracted from the National Health Laboratory Service (NHLS) CDW for the period October 2016 through to June 2017. Data were filtered to include only health facilities within KwaZulu-Natal. The data extract included the absolute CD4 count (< 100 cells/µl) and CrAg result. Additional data variables included the episode number, testing date, health facility location code and description, testing and referring laboratory names, date of birth, patient age (in years) and gender. The testing year and month were derived using the CD4 result review date. Ethics clearance for all CDW data extraction was obtained through the University of the Witwatersrand (M1706108). Data was de-identified at the CDW.

### Data analysis

The CDW data extract were imported into a Microsoft Access table (Washington, United States). Queries were used to prepare the data for analysis. The CDW location code and description were mapped to the District Health Information System (DHIS) health facility descriptions; for example, Addington hospital was mapped to the DHIS organisational unit (OU) 5 short description ‘Addington Hosp’. For each CDW location, in addition to the OU5 facility name, the DHIS province (OU2), health district (OU3), health sub-district (OU4), OU type, latitude and longitude were added to the mapping table. Where a CDW location description was not matched to the DHIS list, it was marked as ‘excluded’ (e.g. Empangeni Prison). The ‘DHIS OU_Type’ was used to identify facility type, for example hospital (tertiary, regional and district), community health centre (CHC), or Primary health care clinic (PHC).

The mapping table was loaded on Microsoft Access and added to the CD4 and CrAg query. The DHIS-mapped list was used to filter the data for legitimate health facilities. The list was also used to report data at the province, district and sub-district health care levels. The final data set was exported as a comma separated values (CSV) file for analysis in Stata SE (Texas, United States) and Microsoft Excel (Washington, United States).

### Provincial CrAg percentage positivity analysis

The CrAg positivity for KwaZulu-Natal was reported as the proportion of CrAg-positive samples divided by all samples (with CD4 count ≤ 100 cells/µl) with the 95% confidence interval (CI) reported. The provincial CrAg positivity was analysed across age ranges, gender, facility types and CD4 test ranges. The CrAg positivity was also assessed monthly to identify changes or patterns over time. A sub-analysis was undertaken for the provincial CrAg-positive samples to determine CD4 test ranges by facility type, gender and age category.

### Health District CrAg percentage positivity analysis

CrAg positivity and prevalence was analysed for the 11 health districts in the province with the 95% CI reported (i.e. Amajuba, eThekwini, Harry Gwala, iLembe, King Cetshwayo, Ugu, uMgungundlovu, Umkhanyakude, Umzinyathi, Uthukela and Zululand). The total CrAg test volumes, number of positive CrAg samples and CrAg positivity was reported for each health district. Both the district CrAg positivity and prevalence are reported with 95% CI.

### Number of CrAg-positive samples by health facility

ArcGIS was used to map the health district CrAg positivity as well as the number of CrAg-positive samples by health facility to identify where patients presented for care. The larger towns in the province were indicated on the map as reference points. The actual number of positive CrAg samples per health facility was reported in five categories: (1) 1–7, (2) 8–20, (3) 21–43, (4) 44–93 and (5) 94–148. Shapefiles were obtained from the municipal demarcation board and the DHIS spatial coordinates were applied for health facilities. District-level percentage of CrAg positivity was reported in four categories each allocated a different colour: (1) ≤ 5.7%, (2) 5.8% – 6.6%, (3) 6.7% – 8.1% and (4) 8.2% – 9.6%. Category ranges were automatically assigned based on actual data.

### Spatial analysis for two districts with the highest CrAg percentage positivity rate

The two districts with the highest CrAg positivity were identified and the respective sub-district and health facility CrAg positivity was reported using ArcGIS. The two district shapefiles were merged using the ArcMap data management toolbox. The percentage of CrAg-positive samples per health facility was reported using five categories: (1) 4.2% – 6.6%, (2) 6.7% – 8.7%, (3) 8.8% – 9.2% and (4) 9.3% – 10.87%. The actual number of positive CrAg samples per health facility was similarly reported in five categories: (1) 1–4, (2) 5–11, (3) 12–28, (4) 29–43 and (5) 44–135. Category ranges were automatically assigned based on actual data.

## Results

### Provincial CrAg percentage positivity analysis

For the reported period, 50 534 CrAg tests were performed (Table 1) after exclusion of 586 samples from the Church of Scotland laboratory (1.2% of provincial volumes). For the entire province, a CrAg positivity of 7.6% (95% CI, 7.3% – 7.8%) was reported. A higher CrAg positivity of 8.0% (7.6% – 8.3%) was reported for males compared to 7.2% (6.8% – 7.5%) for females (*p* = 0.001). In the 16–19 years age group (*n* = 1415), the CrAg positivity was 8.2% (6.8% – 9.8%). A CrAg positivity of 8.4% was reported for both the 40–44 years (7.7% – 9.0%) and older than 49 years (7.7% – 9.1%) age groups. The lowest CrAg positivity was reported for the 15 years and younger group (4.9% [3.9% – 6.2%]) followed by the 20–25 years age group (5.7% [5.0% – 6.6%]). CrAg positivity was the highest for samples requested at hospitals at 10% (9.6% – 10.5%) compared to 6.5% (5.8% – 7.3%) and 5.7% (5.4% – 6.0%) for CHCs and PHCs respectively (*p* = 0.19). CrAg positivity was 12.4% (11.6% – 13.2%) for CD4 counts of ten cells/µl or lower. For the CD4 ranges of 11–29, 30–49 and ≥ 50 cells/µl, a CrAg positivity of 9.9% (9.4% – 10.5%), 7.4% (6.9% – 8.0%) and 5.2% (4.9% – 5.5%) was reported respectively (*p* = 0.21).

**TABLE 1:** KwaZulu-Natal provincial CrAg positivity descriptive statistics from October 2016 to June 2017.

All CrAg tests	CrAg-negative	CrAg-positive	Total	% Total	% CrAg-positive	95% CI	*p*-value
KwaZulu-Natal	46 717	3817	50 534	100	7.6	7.3–7.8	–
**Sex**							
Female	21 774	1679	23 453	46	7.2	6.8–7.5	0.01
Male	23 660	2044	25 704	51	8.0	7.6–8.3	–
Unknown	1283	94	1377	3	6.8	5.6–8.3	–
**Age category (years)**							
≤ 15	1392	72	1464	3	4.9	3.9–6.2	–
16–19	1278	114	1392	3	8.2	6.8–9.8	–
20–24	2972	181	3153	6	5.7	5.0–6.6	–
25–29	6846	514	7360	15	7.0	6.4–7.6	–
30–34	10 108	876	10 984	22	8.0	7.5–8.5	–
35–39	8453	706	9159	18	7.7	7.2–8.3	–
40–44	6219	567	6786	13	8.4	7.7–9.0	–
45–49	3775	269	4044	8	6.7	5.9–7.5	–
> 49	5674	518	6192	12	8.4	7.7–9.1	–
**Facility type**							
CHC	4511	315	4826	10	6.5	5.8–7.3	0.19
PHC	23 715	1440	25 155	50	5.7	5.4–6.0	–
Hospital	18 491	2062	20 553	41	10.0	9.6–10.5	–
**CD4 count range (cells/µl)**							
≤ 10	5982	846	6828	14	12.4	11.6–13.2	0.21
11–29	9358	1032	10 390	21	9.9	9.4–10.5	–
30–49	8814	706	9520	19	7.4	6.9–8.0	–
50–100	22 563	1233	23 796	47	5.2	4.9–5.5	–

CHC, Community health centre; PHC, Primary health care clinic; %, percentage.

### Health district CrAg percentage positivity analysis

CrAg positivity in the 11 districts ranged from 5.7% (Ugu [4.9% – 6.5%]) to 9.6% (Umkhanyakude [8.6% – 10.6%]) with an overall provincial CrAg positivity of 7.6% (7.3% – 7.8%) (Table 2). For the 11 districts, 33.7% of CrAg samples were requested by health facilities in the eThekwini health district (*n* = 17 017). The highest CrAg positivity was reported by the Umkhanyakude (9.6%), King Cetswayo (9.5% [8.7% – 10.4%]) and Zululand (8.1% [7.3% – 8.9%]) districts. The number of CrAg-positive samples per district ranged from 133 (Harry Gwala) to 1304 (eThekwini). Following de-duplication, a provincial CrAg prevalence of 7.5% (7.3% – 7.8%) was reported. CrAg prevalence ranged from 5.5% (4.7% – 6.4%) to 9.7% (8.6% – 10.8%) for the Ugu and Umkhanyakude districts respectively. The highest CrAg prevalence was in the Umkhanyakude (9.7%), King Cetswayo (9.5% [9.0% – 10.8%]) and Zululand (7.9% [7.1% – 8.8%]) districts.

**TABLE 2:** Analysis of district CrAg positivity in KwaZulu-Natal between October 2016 and June 2017 for screened samples and de-duplicated patients de-duplicated patient data.

Health district	CrAg specimen-level data					De-duplicated patient data			
	CrAg test volumes	Total (%)	CrAg-positive samples (n)	% CrAg positivity	95% CI	Patient receiving a CrAg test with a CD4 count < 100 cells/µl	CrAg-positive patients (n)	Cryptococcal antigenaemia prevalence (%)	95% CI
Amajuba	2535	5.0	158	6.2	5.3–7.2	2237	140	6.3	5.3–7.3
eThekwini	17 017	33.7	1304	7.7	7.3–8.1	15 316	1188	7.8	7.3–8.2
Harry Gwala	2011	4.0	133	6.6	5.6–7.8	1838	125	6.8	5.7–8.0
iLembe	2818	5.6	205	7.3	6.3–8.3	2591	183	7.1	6.1–8.1
King Cetshwayo	4882	9.7	466	9.5	8.7–10.4	4427	420	9.5	9.0–10.8
Ugu	3504	6.9	199	5.7	4.9–6.5	3174	175	5.5	4.7–6.4
uMgungundlovu	4481	8.9	292	6.5	5.8–7.3	4032	261	6.5	5.7–7.3
Umkhanyakude	3342	6.6	320	9.6	8.6–10.6	2896	280	9.7	8.6–10.8
Umzinyathi	1929	3.8	141	7.3	6.2–8.6	1716	123	7.2	6.0–8.5
Uthukela	3091	6.1	201	6.5	5.7–7.4	2732	173	6.3	5.4–7.3
Zululand	4924	9.7	398	8.1	7.3–8.9	4290	339	7.9	7.1–8.8

**Total**	**50 534**	**100**	**3817**	**7.6**	**7.3–7.8**	**45 249**	**3407**	**7.5**	**7.3–7.8**

### Number of CrAg-positive samples by health facility

**FIGURE 1:**
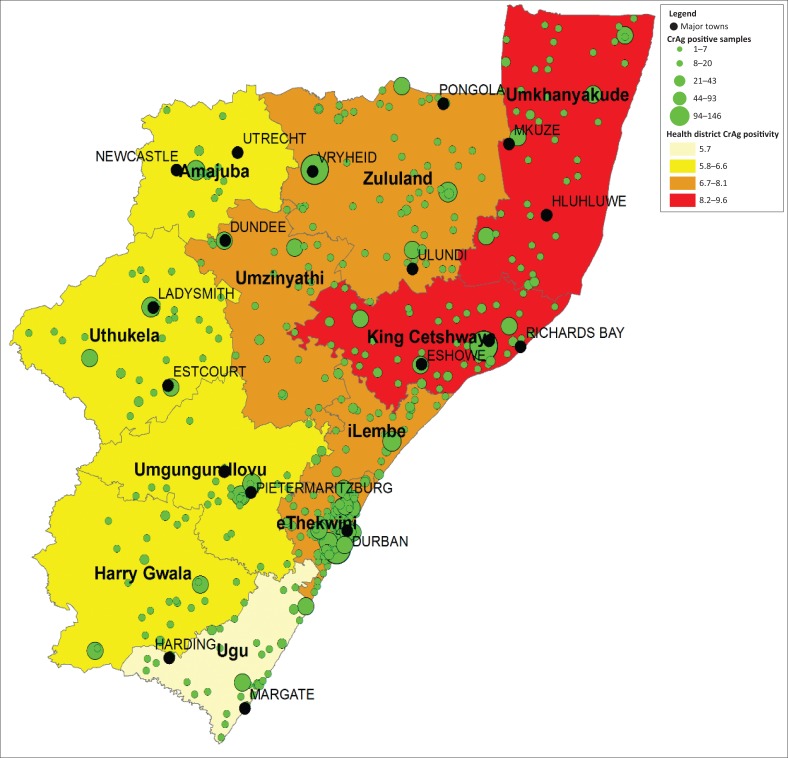
Spatial analysis of the number of positive CrAg samples by health facility reported across five categories

Across the province, 472 health facilities requested a CD4 test for which a reflex CrAg test was performed where the count was ≤ 100 cells/µl. CrAg-positive tests per health facility ranged from 1 to 146 (Figure 1).

A cluster of health facilities (*n* = 120) in close proximity with CrAg-positive results were noted in the eThekwini district (Durban). The eThekwini district CrAg positivity was 7.7%. The number of CrAg-positive samples for this district ranged from one at smaller PHC facilities such as Merebank clinic to 146 at the Prince Mshiyeni Hospital. Two health facilities reported a CrAg positivity above 15%, that is, Mahatma Gandhi hospital (16.7%) and Blue Roof clinic (17.6%).

Smaller clusters of health facilities were noted in the uMgungundlovu (Pietermaritzburg), iLembe, King Cetswayo (Empangeni/Richards Bay), Amajuba (Newcastle) and Ugu (Margate) districts with district CrAg positivity results of 6.5%, 7.3%, 9.5%, 6.2% and 5.7%, respectively. Three health facilities had between 94 and 146 CrAg-positive samples (i.e. Vryheid, Ngwelezana and Prince Mshiyeni Memorial hospitals). There were 11 health facilities that had between 44 and 93 CrAg-positive samples identified, that is, RK Khan, Mahatma Gandhi, Madadeni, Edendale, Stanger, King Edward, Northdale, King Dinuzulu, Ladysmith and Benedictine hospitals as well as the KwaMashu Poly CHC. There were 27 health facilities with 21–43 CrAg-positive samples.

### Health sub-district spatial analysis for two districts with the highest CrAg positivity rate

**FIGURE 2:**
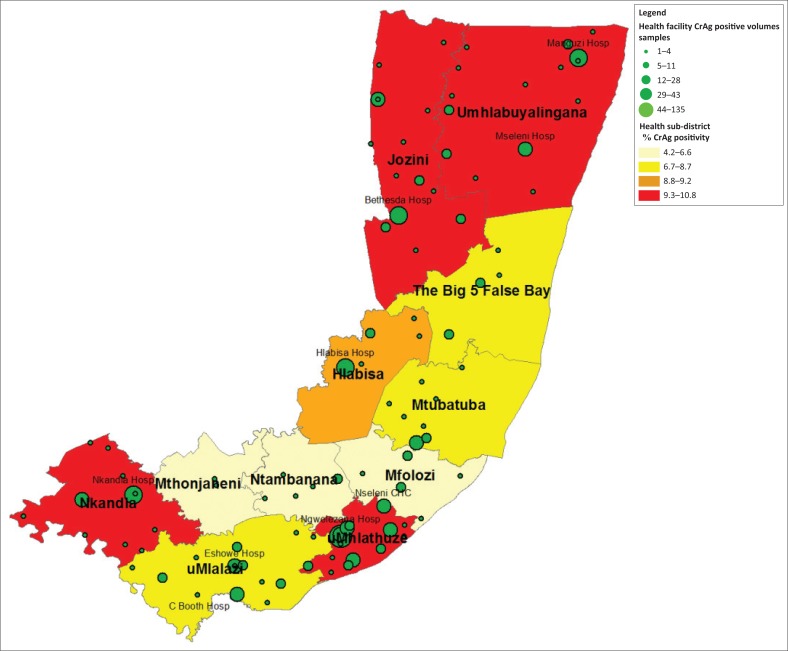
Spatial analysis of the CrAg positivity rate by sub-district for the King Cetshwayo and Umkhanyakude health districts with the number of positive CrAg samples by health facility reported across five categories.

As mentioned, the districts with the highest CrAg positivity rates were Umkhanyakude (9.6%), and King Cetswayo (9.5% [8.7% – 10.4%]). There are five subdistricts in the Umkhanyakude district compared to six subdistricts in King Cetswayo. Across the two districts, CrAg positivity ranged from 4.2% (Mthonjaneni) to 10.8% (Nkandla) (Figure 2). The Jozini, Umhlabuyalingana, uMhlathuze and Nkandla subdistricts were placed in the 9.3% – 10.8% category. Their CrAg positivity rates were 10.2%, 10.0%, 10.5% and 10.8%, respectively. Only the Ngwelezana hospital in the uMhlathuze sub-district was placed in 44–135 CrAg-positive sample category (value of 135). This was followed by four health facilities in the 29–43 CrAg-positive samples (i.e. Hlabisa [43], Manguzi [41], Bethesda [36] and Nkandla [34]). The majority of health facilities (*n* = 93) reported 28 or fewer CrAg-positive samples.

## Discussion

In this study, we describe a CrAg positivity of 7.6% [7.3% – 7.8%] for KwaZulu-Natal. This is the highest provincial CrAg positivity across South Africa.^5^ A statistically significant higher CrAg positivity was reported for males (*p* < 0.05). The majority of CrAg samples (33.7%) in the province were requested by health facilities in the eThekwini health district. District CrAg positivity ranged from 5.5% in Ugu to 9.7% for Umkhanyakude. Three districts reported a CrAg positivity ≥ 7.8% (i.e. Umkhanyakude [9.7%], King Cetswayo [9.5%] and Zululand [7.9%]). Within the Umkhanyakude and King Cetswayo districts, the sub-district CrAg positivity ranged from 4.2% to 10.8%. Four subdistricts within these two districts reported a CrAg positivity between 9.3% and 10.8%.

The proportion of CD4 samples with < 100 cells/µl for the 2014–2015 financial period was used to prioritise the implementation of CrAg screening at the provincial and district levels.^5^ Across South Africa, a proportion of CD4 <100 cells/µl of 9.69% was reported.^5^ The provincial proportion of CD4 samples ≤100 cells/µl ranged from 7.33% for KwaZulu-Natal to 11.82% for Limpopo.^5^ In KwaZulu-Natal, the district proportion of CD4 samples ≤ 100 cells/µl ranged from 5.4% in Umkhanyakude to 9.1% in the Harry Gwala district. Consequently, this province was the last to implement CrAg screening. Given the data reported in this study, it is clear that CrAg prevalence data would have been a better proxy for the order of implementation. Unfortunately, at the time only the proportion of CD4 samples ≤ 100 cells/µl was available.

KwaZulu-Natal has a population of over 11 million people with an HIV prevalence of 16.9% (national prevalence was 12.22%), equating to a HIV-seropositive population of 1.8 million.^6,7^ Over a third (33.5%) of the KwaZulu-Natal population reside in the eThekwini health district (*n* = 3.7 million) followed by uMgungundlovu (9.9%: 1.09 million) and King Cetshwayo (8.8%: 971 thousand).^14^ 34.9% of the population are under 14 years of age,^7^ with 36.7% (*n* = 4 065 052) in the 16 to 34 years of age group. Overall, 71.6% of the provincial population is less than or equal to 34 years of age indicating a young population.^7^ The 2012 Human Sciences Research Council national household survey reported that most districts in the province had an HIV prevalence ranging from 16% to 22% with the exception of Umkhanyakude, Ilembe and eThekwini districts (ranging from 13% to 15%).^15^

An analysis of the HIV-treatment cascade in the province in 2016 revealed that 23% of the HIV-seropositive population did not know their status, that is of the 1.8 million HIV-positive people 1.63 million knew their HIV status.^6^ By 2016, 1.06 million people were on ART (87% of HIV-positive people that know their status).^6^ Johnson et al. assessed the provincial progress towards the 90–90–90 targets and reported a provincial ART coverage of 62% compared to 57% nationally.^16^ To improve ART coverage, the province recently adopted the Universal Test and Treat (UTT) strategy in which all HIV-seropositive individuals receive ART regardless of CD4 count.^6^ The goal of the UTT strategy is to reduce the incidence of HIV infections.^6^ The CrAg burden revealed in this study is possibly linked to the significant burden of patients still to be initiated on ART or who disengage from HIV care.

This study has demonstrated the inherent value of laboratory data to indicate how patients use and access services, to understand the burden of disease as evidenced in the study results and facilitate the identification of hotspots to guide programmatic interventions. This intrinsic value can be utilised at the national, provincial, district, sub-district and facility levels and even at the patient level and linked to service delivery or to a specific health programme. This is not possible with aggregate data systems. A good example is the results for action report that can deliver a list of babies that are HIV-positive to the district manager to ensure that they are linked to care.^17^ The data are compressed and password-protected to maintain confidentiality.^17^ To receive the results for action reports, healthcare workers are required to apply online using the CDW self-service portal.^17^ Additionally, the laboratory data can provide indirect information about healthcare services that are being accessed by assessing what are being requested and from which health facility (based on geographic location, that is latitude and longitude). The use of spatial tools allows for analysis at a glance by presenting the information in a way that makes it easy to visualise hotspots and further identify how homogenous or heterogeneous CrAg positivity is, for example. Aside from offering a broader public health perspective at the national level, due to linkage to specific geographical identifiers, the analysis enables drilling down to an individual health facility level to directly affect individual patient management or intervention.

Specifically, in this study we have illustrated, with the outcomes reported here, how specimen-level laboratory data can be used to assess a laboratory test, CrAg, and establish the positivity at the provincial, district, sub-district and even at health facility levels. There was no bias or predominant area with higher testing numbers; we have shown that CrAg screening coverage appears to be widely (and universally) spread across the province, with 473 health facilities linked to at least one positively identified CrAg sample. The information also suggests that the implementation of the national reflex CrAg testing programme is facilitating CrAg testing from all health facilities through a CD4 test request to their local laboratory. The analysis has further revealed that CrAg positivity is not uniformly distributed at either the district or sub-district-level, with some districts demonstrating a much higher positivity; to effect meaningful clinical and programmatic impact at facility level, further investigation is needed to assess the positivity and assess whether a similar pattern could be anticipated at facility level as well. In this study we also reported the number of CrAg-positive samples at the health facility level. This approach enables programme managers to identify health facility hotspots to focus programme or clinical interventions that would assist in the identification of patients who are more likely to have cryptococcal disease before they become sick.

Future directives and initiatives requiring coordination and collaboration by both the NHLS and the national, regional and provincial departments of health are needed, to ensure that patients are linked to care. Aside from the existing ‘results for action’ report of CrAg-positive patients that is issued at sub-district or facility level by the NHLS for individual patient follow-up, a more generalised collated data report about the volumes of patient accessing specific services, and how sick these patients are, is necessary. This report could be used to ensure that the original aim of the CrAg screening initiative is realised and that there is meaningful infrastructure and systems streamlined so that the follow-up of HIV-seropositive patients who additionally require antifungal treatment when diagnosed with early cryptococcal disease, receive this treatment.

### Limitations

Information about patient outcomes where a positive CrAg result was reported were not available. There is, however, currently a planned collaboration between the National Institute for Communicable Diseases and the National Department of Health to undertake a prospective study. The purpose is to understand the outcomes of the SA CrAg screening initiative by following a CrAg-positive cohort from diagnosis to treatment. This will help to address some of the additional questions that cannot be answered solely with laboratory data. Additional questions that can be answered may be the loss to follow up, whether patients positively identified through the screening initiative receive treatment and what their clinical outcomes are. All CD4 samples tested for CrAg at the Church of Scotland laboratory were excluded from this analysis.
